# Interventional Cardio-Oncology: Unique Challenges and Considerations in a High-Risk Population

**DOI:** 10.1007/s11864-023-01110-2

**Published:** 2023-06-10

**Authors:** Orly Leiva, Usman Alam, Isaac Bohart, Eric H. Yang

**Affiliations:** 1 Division of Cardiology, Department of Medicine, New York University Grossman School of Medicine, New York, NY USA; 2 Department of Medicine, New York University Grossman School of Medicine, New York, NY USA; 3 Division of Cardiology, Department of Medicine, UCLA Cardio-Oncology Program, University of California at Los Angeles, 100 Medical Plaza, Suite 630, Los Angeles, CA 90095 USA

**Keywords:** Cardio-oncology, Percutaneous coronary intervention, Transcatheter valve replacement, Atrial fibrillation, Left atrial appendage occlusion, Atrial fibrillation ablation

## Abstract

Patients with cancer are at risk of developing cardiovascular disease (CVD) including atherosclerotic heart disease (AHD), valvular heart disease (VHD), and atrial fibrillation (AF). Advances in percutaneous catheter-based treatments, including percutaneous coronary intervention (PCI) for AHD, percutaneous valve replacement or repair for VHD, and ablation and left atrial appendage occlusion devices (LAAODs) for AF, have provided patients with CVD significant benefit in the recent decades. However, trials and registries investigating outcomes of these procedures often exclude patients with cancer. As a result, patients with cancer are less likely to undergo these therapies despite their benefits. Despite the inclusion of cancer patients in randomized clinical trial data, studies suggest that cancer patients derive similar benefits of percutaneous therapies for CVD compared with patients without cancer. Therefore, percutaneous interventions for CVD should not be withheld in patients with cancer, as they may still benefit from these procedures.

## Introduction

Malignancy and cardiovascular disease are the two leading causes of death in the developed world [1]. However, advances in cancer diagnostics and therapies have improved cancer-specific survival and outcomes [2, 3]. Indeed, cardiovascular disease is an important contributor to long-term mortality among cancer survivors [3]. Emerging data suggests that cancer and cardiovascular disease share similar risk factors and underlying pathophysiology including comorbidities (smoking, age, obesity, diabetes) and inflammation [4••]. Additionally, cancer-directed therapies themselves may accelerate the development of cardiovascular disease [4••].

Cardiovascular disease is a broad term that encompasses a heterogeneous group of disorders ranging from coronary artery disease (CAD), valvular heart disease, cardiomyopathy, arrhythmias, and their complications. Advances in transcatheter and minimally invasive treatment modalities have improved outcomes in many cardiovascular diseases including, but not limited to, percutaneous coronary intervention (PCI) for acute myocardial infarction (AMI) and CAD, transcatheter valve replacement for aortic stenosis (TAVR), and left atrial appendage occlusion (LAAO) for stroke prevention in atrial fibrillation (AF) [5•, 7,6•, ]. However, patients with active cancer have been historically excluded from device and intervention trials. Additionally, patients with cancer are often less likely to have procedures performed despite still having possible benefit [8, 9]. Therefore, in the present report, we will review common pathophysiology between cancer and cardiovascular disease and data on transcatheter and minimally invasive modalities commonly used to treat them.

## Atherosclerotic heart disease and percutaneous coronary intervention

Patients with cancer are at increased risk of arterial thrombosis, including myocardial infarction (MI), compared with patients without cancer. In one study, risk of MI was highest within the first month of cancer diagnosis (HR 7.3, 95% CI 6.5 to 8.2) [10]. Patients with lung (HR 10.1, 95% CI 8.0–12.8), colorectal (HR 12.6, 95% CI 9.5–16.7), pancreatic (HR 13.9, 95% CI 7.7–25.0), and gastric cancer (HR 11.0, 95% CI 5.3–22.6) had the highest risk of MI compared with patients without cancer [10]. Cancer and atherosclerotic heart disease share common pathophysiology and risk factors including chronic inflammation [4••]. Indeed, anti-inflammatory therapies including anti-IL1β inhibition and colchicine have been studied in randomized controlled trials (RCTs) in patients at risk for and with coronary artery disease (CAD) and have shown reductions in cardiovascular events [11–13]. While common pathophysiology and risk factors are shared between cancer and atherosclerosis, cancer therapy itself may exacerbate and accelerate the development of atherosclerosis. Radiation therapy has been shown to accelerate coronary artery atherosclerosis and increase the risk for ischemic heart disease [14, 15]. Conventional chemotherapy has also been associated with accelerated atherosclerosis and myocardial infarction [4••]. Inhibition of vascular endothelial growth factor (VEGF) signaling (either via neutralization via antibodies or indirectly via tyrosine kinase inhibition), commonly used in the treatment of various malignancies, has also been associated with increased risk of myocardial infarction [16]. Immune checkpoint inhibitors (ICIs) have been associated with accelerated atherosclerosis and plaque progression [17•, 18].

Percutaneous coronary intervention (PCI) is indicated for revascularization among patients presenting with acute coronary syndrome (acute myocardial infarction, unstable angina), or for selected stable patients with refractory angina despite maximally tolerated medical therapy [19]. Among patients undergoing PCI, the prevalence of patients with cancer has increased in the past 20 years with the fastest rate of increase being of lung cancer patients [20••]. In a large retrospective study of over 6 million inpatient PCI procedures, patients with cancer were associated with increased risk of in-hospital mortality and bleeding [20••]. Patients with lung cancer were at the risk of in-hospital mortality with an odds ratio (OR) of 2.81 (95% confidence interval 2.37–3.34), while patients with colon cancer had the highest risk of bleeding (OR 3.65, 95% CI 3.07–4.35) compared with patients without cancer [20••]. In another study large study of 6.5 million patients hospitalized for acute myocardial infarction, patients with cancer had increased rates of in-hospital death (11.1% vs 5.7%), bleeding (18.4% vs 8.8%), and were less likely to undergo PCI (27.1% vs 43.9%) compared with patients without cancer [21]. However, among patients with cancer who presented with ST-elevation myocardial infarction (STEMI), patients treated with PCI were associated with lower risks of in-hospital mortality that were comparable to patients without cancer [22]. Additionally, PCI was associated with lower total hospitalization costs among patients with cancer independent of length of stay [23].

PCI is often performed in conjunction with intracoronary physiologic testing (including fractional flow reserve) and/or intravascular imaging, including intravascular ultrasound (IVUS) and optical coherence tomography (OCT). Studies have suggested that the use of IVUS or fractional flow reserve (FFR) improve post-PCI outcomes by improving lesion selection and optimization of stent sizing and deployment [24–26]. In a study of patients with cancer undergoing PCI, FFR was utilized in 3.4% of patients and was associated with lower in-hospital mortality and length of stay [27]. Case reports have described successful use of intracoronary imaging in patients with cancer; however, larger studies are lacking [28, 29].

Management of patients with cancer post-PCI can be challenging [30]. Patients who undergo PCI require dual antiplatelet therapy (DAPT) for an extended amount of time which increases risk of bleeding [31]. However, optimal duration of DAPT is unclear in patients with cancer given that different types of cancer have a heterogeneous risk of bleeding and patients with cancer are often excluded for randomized clinical trials. However, 1 month of DAPT followed by single anti-platelet therapy (SAPT) in patients at high risk of bleeding was associated with decreased bleeding compared with extended DAPT (3 months) with no increase in thrombotic events [32••]. Therefore, among patients with cancer at high risk, 1 month of DAPT may be reasonable. Further studies of long-term DAPT is needed among patients with cancer.

## Valvular disease and cancer

Cancer therapy–induced cardiotoxicity not only causes cardiomyopathy or accelerated atherosclerosis, but can also exacerbate valvular heart disease. Valvular heart disease, particularly aortic stenosis (AS), is a condition that increases in prevalence with age. Cancer therapeutics, most notably thoracic radiotherapy (XRT), has been associated with progression and development of AS. It is estimated that 37 to 81% of patients who received thoracic XRT develop valvular disease [33, 34]. Additionally, among patients with AS patients who received XRT had increased risk of long-term mortality compared with patients without a history of XRT [34]. The risk of developing AS and aortic valve fibrosis increases with XRT exposure in a dose-dependent manner, with patients who had received greater than 30 Gray (Gy) of radiation have the greatest risk [35, 36].

Symptomatic, and in some cases asymptomatic, severe AS is an indication for aortic valve replacement (AVR) [37]. AVR can be performed via surgery (SAVR) or minimally invasive transcatheter (TAVR) techniques. Several randomized control trials have shown TAVR to be either non-inferior or superior to SAVR for severe AS [38–40]. Despite the mortality benefit of AVR in severe AS, one study of 3815 patients with severe AS suggested that patients with malignancy are less likely to undergo AVR [41]. However, several studies have suggested that patients with cancer who had undergone TAVR have similar outcomes compared with patients without cancer [42–45,44•, ]. In a single-center study of 477 patients who underwent TAVR (91 with prior cancer), there was no difference in all-cause mortality after a mean follow-up of 851 days. However, cancer therapy administered within 12 months of TAVR was associated with increased risk of death (HR 4.38, 95% CI 1.14–16.77) [42]. In another registry of patients who had undergone TAVR, patients with active cancer had similar 30-day and midterm outcomes compared with patients without cancer though metastatic cancer was associated with increased risk of late mortality (after 30 days) [46]. These results were in agreement with an international registry study of 222 cancer patients who underwent TAVR. This study demonstrated that patients with cancer, when compared to 2522 non-cancer controls, had similar 30-day mortality rates compared with patients without cancer. Additionally, early stage cancer had similar 1-year survival compared with non-cancer patients. However, patients with stage 3 or 4 cancer had increased risk of 1-year mortality; they also observed out of the 85% of patients in the registry that were alive at 1 year, one-third were in remission and/or were considered cured from cancer [47]. Another institutional retrospective found frailty to be more associated with worse outcomes than cancer history [45]. Prior chest XRT not only increases risk of the development of severe AS, but also increases risk of surgery. In a pooled registry study of patients who underwent TAVR, patients with prior chest XRT had no difference in all-cause death or stroke at 2 years compared with patients without prior chest XRT (30.7% vs 27.0%, HR 1.08, 95% CI 0.66–1.77) [48•]. Additionally, patients with prior chest XRT had similar rates of complications, including myocardial infarction, vascular complications, acute kidney injury, or new pacemaker implantation after TAVR [48•].

In addition to AS, patients with cancer may also develop mitral valve regurgitation (MR). The primary mechanisms of MR in cancer patients include radiation-induced valvular damage and inability for mitral valve leaflets to close due to left ventricular dilation due to cardiomyopathy [3]. For patients with symptomatic MR, valve repair or replacement is recommended in most patients [37]. In the past decade, transcatheter mitral valve edge-to-edge repair (TEER) has been developed. In randomized trials of severe degenerative MR, TEER has been shown to have similar outcomes and better procedural safety compared with surgical mitral valve repair or replacement [49]. A study of the National Inpatient Sample (NIS) of patients undergoing TEER or surgical mitral valve repair or replacement with a history of cancer suggested that patients with cancer have similar rates of in-hospital stroke or mortality of patients undergoing TEER compared with surgery [50]. In a separate study of patients undergoing TEER with or without cancer, patients with cancer had similar all-cause mortality and all-cause hospitalizations at 1 and 12 months compared with patients without cancer [51]. These studies suggest that TEER is safe and efficacious in patients with cancer and a history of cancer should not preclude evaluation of this treatment modality.

These findings call for further study in determining which cancer patients benefit from transcatheter therapies, due to the wide heterogeneity of malignancy type, cancer treatments, prognosis, and frailty seen in the cancer population.

## Atrial fibrillation and left atrial occlusion in patients with cancer

Atrial fibrillation (AF) is common among patients with cancer and patients with cancer may have an increased incidence of AF compared with the general population [52]. Furthermore, the development of AF was an independent risk factor for all-cause and cardiovascular mortality in patients with cancer [53]. Risk scores for arterial thromboembolism in AF, such as CHA_2_DS_2_-VASC, do not include cancer, although cancer may affect the risk this outcome [54]. The association between AF and malignancy may be attributed to overlapping risk factors for both conditions, increased chronic inflammation and adverse effects of cancer treatments [4••]. Off-target effects of cancer therapeutics (including conventional chemotherapies and targeted therapies) may increase the risk of AF in patients with cancer [55•].

The management of AF depends on underlying etiology of AF and symptom burden and includes antiarrhythmic therapies, catheter-based ablation, and left atrial appendage occlusion devices (LAAOD) for thromboembolism prevention in patients who cannot tolerate anticoagulation [56]. Catheter-based ablation may offer improved outcomes among patients with AF. In the Early Treatment of Atrial Fibrillation for Stroke Prevention Trial (EAST-AFNET 4), patients with new-onset AF who were assigned to early rhythm control rather than usual care experienced decreased risk of a composite of cardiovascular mortality, stroke, and hospitalization with heart failure or acute coronary syndrome (HR 0.79; 95% CI 0.66–0.94), suggesting that rhythm control may be superior to rate control for properly selected patients with AF [57]. Rhythm control strategies employed in the trial included both antiarrhythmic mediations and ablation [57]. AF ablation techniques have improved substantially in recent years, and multiple trials have demonstrated superiority of pulmonary vein isolation (PVI) using cryoablation over antiarrhythmic medications for prevention of recurrent atrial arrhythmias [58, 59]. However, data on ablation outcomes and safety for patients with cancer is limited [60, 61]. In one observational study, the rates of complications and arrhythmia-free survival at 12 months did not differ significantly between 70 patients with past or present cancer undergoing cryoballoon-based PVI as compared to 70 propensity score–matched controls [62]. Notably just 8/70 patients in the study had active cancer when undergoing ablation [62]. Cancer survivors were also noted to experience an elevated rate of clinically significant bleeding after radiofrequency ablation for AF (OR 3.60; 95% CI 1.02–12.73) [63]. In another study, patients with a history of breast cancer were at elevated risk of AF recurrence 1 year after ablation as compared to propensity score–matched controls (OR 2.68, 95% CI 1.05-6.86), but no difference in the rate of procedural complications was observed [64]. Additionally, prior mediastinal radiation therapy was identified as an independent risk factor for AF recurrence after ablation (aOR 4.79; 95% CI 1.34–17.1) [64]. However, another study of 502 patients (251 patients with cancer), patients with cancer had similar rates of AF recurrence at 12 months and repeat AF ablation [65]. Additional data are needed in order to further elucidate risks of complications and treatment failure among patients with active cancer undergoing ablation for AF.

Given the associations between cancer and both bleeding and thrombosis, the decision to initiate anticoagulation in patients with cancer and AF may be challenging [66]. The development of percutaneous left atrial appendage occlusion (LAAO) techniques may be beneficial to reduce the risk of thromboembolism in patient populations at high risk of bleeding with anticoagulation [67]. However, few studies have examined the efficacy and complication rate of LAAO in patients with cancer. In a population-based study of 15,895 patients in Germany undergoing percutaneous LAAO, cancer was an independent predictor of in-hospital mortality (OR 2.49; 95% CI 1.00–6.20) [68]. In another study of 15,399 patients in the USA undergoing percutaneous LAAO, there was no difference in the composite of in-hospital death, ischemic stroke/transient ischemic attack (TIA), systemic embolism, bleeding requiring blood transfusion, pericardial effusion treated with pericardiocentesis or surgery, and removal of embolized device across groups of patients with active cancer, a history of cancer, or no cancer history [69•]. However, a higher risk of stroke/TIA during admission was associated with active cancer (aOR 3.06; 95% CI 1.17–8.01) but not with prior cancer [69•]. In contrast to the prior study, no difference in in-hospital mortality based on cancer status was noted [69•]. There was also no difference in readmission for TIA or ischemic stroke within 30 or 180 days across groups, suggesting acceptable short-term efficacy of LAAO for prevention of these outcomes in patients with cancer [69•]. In another study, the rate of ischemic stroke at a follow-up of 1.8 ± 1.1 years among 55 patients with prior or current cancer undergoing LAAO was 3.6% [70]. Although no comparison group without cancer was included in this study, this event rate is similar to the rate of stroke and TIA of 2.37 events per 100 patient-years in the LAAO group in the PRAGUE-17 trial [71]. Another published abstract reported that among patients undergoing LAAO, those with cancer experienced higher inpatient mortality (0.65% vs 0.14%, *p* = 0.007) but similar 30-day readmission rates (10.0% vs 9.1%, *p* = 0.34) [72]. In a single-center study of patients with nonvalvular AF who underwent LAAO with or without cancer, there was no difference in risk of ischemic stroke (HR 0.44, 95% CI 0.10–1.97), bleeding (HR 0.71, 95% CI 0.28–1.86), or death (HR 1.39, 95% CI 0.73–2.64) among cancer patients compared to without cancer [73]. Data on the long-term efficacy of LAAO in patients with cancer are limited, and further studies are needed to investigate whether LAAO provides benefit in this high-risk patient population.

## Heart failure, shock, and temporary mechanical circulatory support in patients with cancer

Heart failure and its complications, including cardiogenic shock, uncommonly do not occur in patients with cancer [4••]. As patients with cancer live longer due to advances in therapies, cardiovascular disease and heart failure are becoming an increasing source of morbidity and mortality in this patient population. Cancer-specific therapies may also have cardio-toxic effects that may lead to acute heart failure (HF) and cardiogenic shock including immune checkpoint inhibitors (ICI), anthracyclines, and tyrosine kinase inhibitors (TKIs).

Immune checkpoint inhibitors have become common and effective therapies for a variety of cancers and have revolutionized cancer therapies. However, immune-related adverse events, including myocarditis, have been described to occur in patients receiving ICI therapies and may carry significant morbidity and mortality [74]. In one meta-analysis of 63 randomized clinical trials (RCTs) of ICI, there was an incidence of myocarditis of 3.2 per 1000 patients [75]. The presentation of ICI myocarditis varies from asymptomatic to fulminant myocarditis with rapid progression to cardiovascular collapse, unstable arrhythmias, or heart failure symptoms [76]. The median onset of ICI myocarditis has been described to be between 27 and 34 days but may occur as late as 1 year post-ICI initiation [76, 77]. A high degree of suspicion for ICI myocarditis is needed, given prompt recognition is essential to initiate timely therapy and reduce risk of fulminant myocarditis and death, which may be as high as 40 to 50% [77]. The treatment of ICI myocarditis involves immunosuppression to quell the overactive immune system and limit end-organ damage [78]. Earlier initiation of steroids and higher steroid dosing have been associated with decreased adverse events, with less than 24 h having the best outcomes [76]. Conventional chemotherapies, especially anthracyclines (including doxorubicin, daunorubicin, idarubicin, epirubicin, mitoxantrone) are associated with cardiotoxicity including cardiomyopathy and arrhythmias [79]. Another conventional chemotherapy associated with cardiotoxicity and HF is 5-fluorouracil (5-FU). After anthracyclines, 5-FU is the second most common drug associated with cardiotoxicity [80]. Patients with 5-FU cardiotoxicity may present with acute chest pain, myocardial infarction, heart failure, arrhythmias, and sudden cardiac death [80].

Among patients with heart failure and cardiogenic shock, the use of acute mechanical circulatory support (MCS) is utilized to stabilize hemodynamically unstable patients as a bridge to recovery or other advanced therapies (durable MCS, transplant, etc.) [81]. The most commonly used MCS devices include intra-aortic balloon pump (IABP), Impella, extracorporeal membrane oxygenation (ECMO), tandem heart, and CentriMag. Literature surrounding outcomes and characteristics of patients with cancer treated with other forms of MCS (including IABP and Impella) is lacking despite the increased use of both modalities in the management of cardiogenic shock. However, outcomes of patients with cancer managed with ECMO has been described in the literature.

There are two different types of ECMO, venoarterial (VA) and venovenous (VV). While both types of ECMO provide respiratory support, VA-ECMO also provides hemodynamic support and is therefore used most frequently in patients with cardiogenic shock. Case reports have described successful use of MCS, particularly VA-ECMO, as a bridge to recovery in ICI myocarditis [82–84]. However, the decision to escalate to MCS in cancer patients is a complex one and requires multidisciplinary input, given the heterogeneous stages and prognosis of malignancies that these patients may present in. For instance, a patient with end-stage malignancy on palliative treatments may not benefit from MCS due to a lack of a realistic destination to bridge to (i.e., heart transplant, left ventricular assist device) due to a poor prognosis and limited survival with their cancer. However, respiratory compromise for a variety of reasons is seen in patients with cancer, and therefore, VV-ECMO has been studied in cancer patients in these scenarios. In a study of 297 cancer patients from 19 European hospitals who underwent VV-ECMO, the 60-day overall survival rate was 27% and severe bleeding was seen in 38% of patients [85]. Patients with hematologic malignancy patients had increased risk of bleeding compared with solid malignancies (44% versus 33%) [85]. Another study of patients with cancer treated with ECMO showed lower survival in-hospital (13% vs 38%) and 6-month survival (3% vs 26%) rates among patients with hematologic malignancies compared with solid tumors [86]. In a small, single-center study of 23 patients with hematologic malignancies on ECMO (14 on veno-arterial ECMO and 9 on VV-ECMO), there was a significant rate of in-hospital mortality with 91% of patients dying after a median ECMO duration of 105 h [87]. A meta-analysis of 13 observational studies (including 422 patients with hematologic malignancies) showed a pooled in-hospital mortality rate of 79% [88]. These studies suggest that patient selection is important for the determination of ECMO utilization in patients with cancer, particularly those with hematologic cancers.

## Venous thromboembolism, endocarditis and cancer

### Venous thromboembolism

The link between cancer and thrombosis was first described by Trousseau in 1865, and a clear link between cancer and hypercoagulability has been established [89]. Since this initial observation, several studies have corroborated that cancer patients have an increased prevalence of venous thromboembolism (VTE) which is estimated to be between 5 and 10% [90]. The pathophysiology of cancer-associated VTE is multifactorial and include tumor-specific risk factors (tumor histology, stage, mutations, etc.), chemotherapy, increased venous stasis from immobility, surgery and increased inflammation [89]. Among patients hospitalized for PE, patients with cancer had increased risk of in-hospital death compared with patients without cancer (11.8% vs 6.6%, *p* < 0.001) [91]. Anticoagulation is the first-line therapy for patients with PE, with thrombolytic therapy being reserved with patients with hemodynamic instability [92, 93]. Catheter-based therapies (CBTs), including catheter-directed thrombolysis (CDT) and percutaneous mechanical thrombectomy (MT), have been developed as a therapeutic option for patients with PE. Improvements in surrogate outcomes with CBT, including right ventricular (RV) dysfunction and hemodynamic parameters, have been demonstrated in small, single-armed trials and registries in patients with intermediate and high-risk PE [94–98]. Current guidelines recommend CBT for patients with high-risk PE at high bleeding risk or have contradictions to or failed systemic thrombolysis [99, 100]. Several case reports have described the successful use of mechanical or aspiration thrombectomy in cancer patients [101, 102]. However, robust data regarding outcomes of patients with cancer undergoing CBT are scarce and in need of further investigation.

### Endocarditis

Cancer patients are at increased risk for both infective endocarditis and non-bacterial thrombotic endocarditis (NBTE) [103]. Patients with cancer are at increased risk of infective endocarditis due to several factors including indwelling central venous catheters, hospital-based procedures, and immunosuppression from cancer-directed therapies [103]. Additionally, compromise of the gut wall integrity either by tumor invasion directly or chemotherapy-induced damage often leads to bacterial translocation and increases the risk of infective endocarditis [104]. Patients with cancer and infective endocarditis have worse outcomes compared with patients without cancer. One study showed increased in-hospital (34.8% vs 25.8%, *p* = 0.012) and 12-month mortality (47.8% vs 30.9%, *p* < 0.01) among infective endocarditis patients with cancer compared with patients without cancer [103]. Current guidelines recommend early surgical intervention for infective endocarditis in patients with valvular dysfunction causing heart failure, endocarditis by *Staphylococcus aureus*, fungal or other resistant organisms, structural complications (including heart block, annular or aortic abscess), or persistent bacteremia despite adequate antibiotics [37]. Despite these recommendations, patients with cancer are less likely to undergo surgery if indicated compared with patients without cancer, which may be, at least in part, due to worse overall prognosis and frailty in patients with cancer [103, 105]. The development of percutaneous, vacuum-based aspiration devices including the AngioVac system (AngioDynamics, Latham, NY), has provided a minimally invasive technique for the off-label management of infective endocarditis in patients at prohibitive surgical risk [106]. While percutaneous vacuum-based aspiration of infective endocarditis is most often performed for right-sided lesions given the risk of embolism and stroke, mitral valve interventions have been described in the literature using transseptal puncture and cerebral embolization protection techniques [107]. Percutaneous vacuum-based aspiration may offer a less invasive treatment for patients with cancer and infective endocarditis, though high-quality data are sparse. However, several case studies have described successful vacuum-assisted embolectomy from the tricuspid valve or right atrial wall in patients with cancer patients [108–111]. Additionally, one small study of 44 patients (20 with active malignancy) suggested that vacuum-assisted thrombectomy may be safe in patients with cancer [112]. Further investigation of whether percutaneous thrombectomy affects short-term and long-term outcomes is warranted.

## Conclusions

**Fig. 1:**
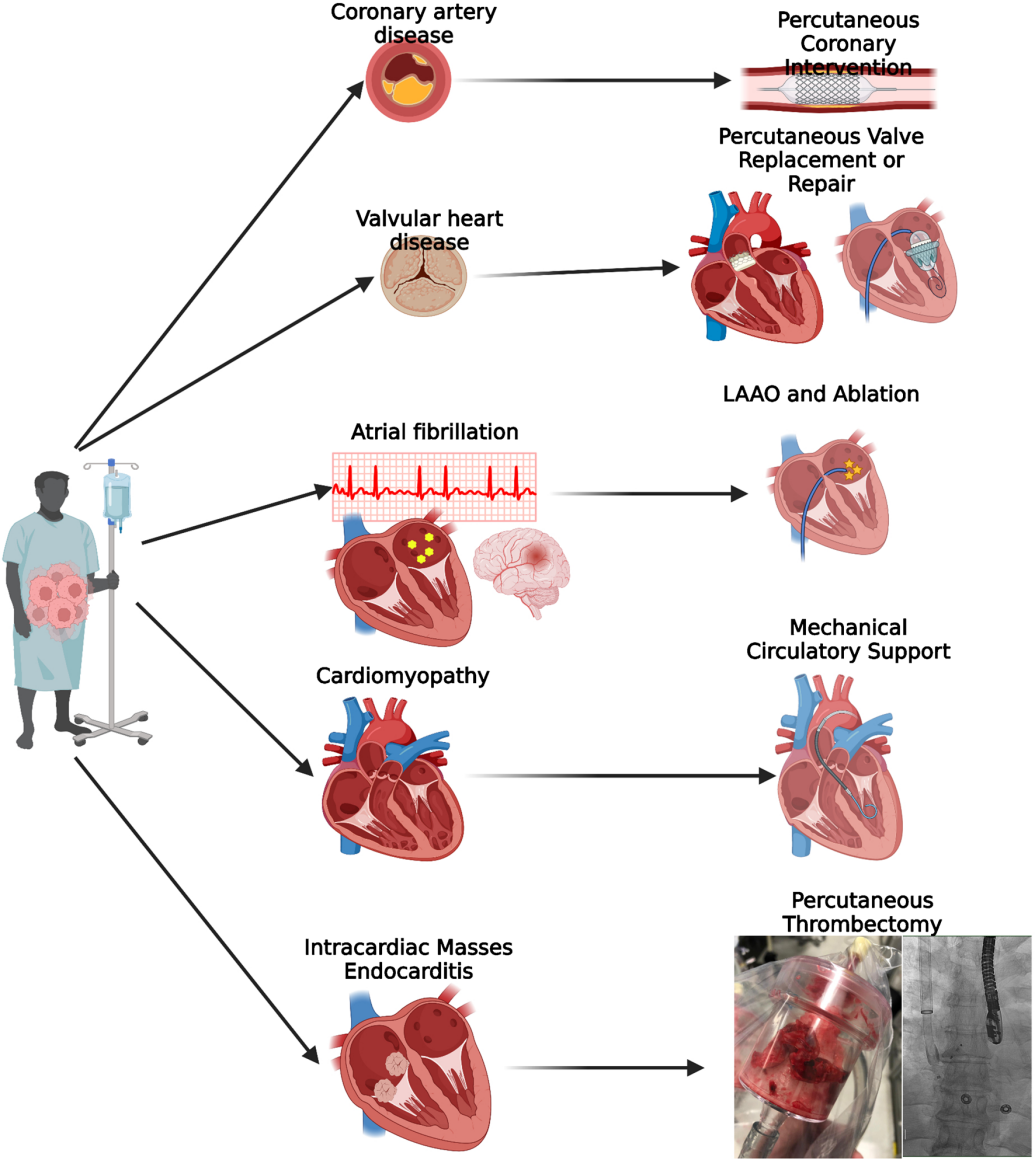
Patients with cancer are at risk of developing a variety of cardiovascular disease including coronary artery disease (CAD), valvular heart disease (VHD), atrial fibrillation (AF), heart failure and cardiogenic shock and intracardiac masses. Percutaneous interventions have been developed for the treatment of these diseases and are options for patients with and without cancer. Figure created using Biorender.com.

Innovations in cancer therapies have improved cancer-specific outcomes at the expense of increased cardiovascular risk in cancer survivors. However, advances in percutaneous and minimally invasive structural heart procedures have expanded therapeutic options for several cardiovascular diseases (Fig. 1). Additionally, these new procedures offer patients with cancer a safer alternative to conventional surgery. However, there is a paucity of outcome and safety data in several percutaneous cardiac procedures, including MCS and PE thrombectomy, that merit further investigation. Further investigation is needed in order to define patient populations that would benefit most from these interventions and to reduce complications and adverse events. Prospective registries of patients with cancer undergoing interventional cardiology procedures and inclusion of cancer status and characteristics among existing registries would be beneficial for investigating and improving outcomes in this patient population.
